# Historical dataset of mills for Galicia in the Austro-Hungarian Empire/southern Poland from 1880 to the 1930s

**DOI:** 10.1016/j.dib.2021.107709

**Published:** 2021-12-13

**Authors:** Krzysztof Ostafin, Magdalena Jasionek, Dominik Kaim, Anna Miklar

**Affiliations:** Jagiellonian University, Faculty of Geography and Geology, Institute of Geography and Spatial Management, Gronostajowa 7, Kraków 30-387, Poland

**Keywords:** Watermill, Windmill, Sawmill, Historical GIS, HGIS, Galicia, Central Europe

## Abstract

In this article, we present the dataset of mills from 1880 and 1920s–1930s in the area of the former Galicia (78,500 km^2^), now in Ukraine and Poland. The data was obtained as a result of manual vectorisation from 162 map sheets at scales of 1:115,200 and 1:100,000, according to the map legends. We found 4022 mill locations for 1880 and 3588 for the 1920s–1930s. We present them as vector points in shapefile, GML, GeoJSON, KML formats with attributes for seven types of mills for 1880 and ten types of mills for 1920s–1930s, and mills counted in a 10 km grid.

The data can be used in economic, demographic and environmental reconstructions, e.g. to estimate historical anthropopressure related to settlement, agriculture and forestry. Mills are often associated with river structures such as floodgates, dams, and millraces and therefore they are a good example of human interference in river ecosystems. They can also be one criteria for identifying areas where the local population used traditional environmental knowledge. It can be useful for a contemporary assessment of the environment's suitability for devices using renewable energy sources. Finally, the data on the remains of former mills is suitable for the protection of cultural heritage sites that are technical monuments related to traditional food processing and industry.

**Specifications Table**

**Table:** 

Subject	Geography
Specific subject area	Geography, History, Geographical Information System, Engineering
Type of data	Points vector data layer Grid 10 km
How the data were acquired	Data was acquired by manual vectorisation from 19th and 20th century maps
Data format	Raw: points vector data, shapefile, GML, GeoJSON, KML/KMZ Filtered: Grid (areas) vector data, shapefile, GML, GeoJSON, KML/KMZ
Description of data collection	The dataset includes the location of former mills for the historic region of Galicia (78,500 km^2^), currently located in Ukraine and Poland. The data was obtained from Austrian and Polish military maps with scales close to 1:100,000. These maps show the location of economic facilities accurately and in detail.
Data source location	https://tiles.arcgis.com/tiles/JUmUgCafSWdOK8hF/arcgis/rest/services/Kummersberg_1880/MapServer?f=jsapi&cacheKey=8ffcdcf9c5e74422 http://igrek.amzp.pl/mapindex.php?cat=WIG100
Data accessibility	Repository name: Mendeley Data Data identification number: 10.17632/8h9295v4t3.2 Direct URL to data: https://data.mendeley.com/datasets/8h9295v4t3/2

**Value of the Data**

• Data on the location of the mills, with different types of propulsion and functions, is obtained for a large area in Central Europe from consistent and detailed sets of maps over two time periods.
• The vectorised points of the mills were verified based on counts with sources from statistical lists. The census data on the mills did not allow them to be easily localised in the region. Thanks to the geometric data, it is possible to verify them on both sides and with other sources.
• The data is available in an open GIS formats, easy to visualise and use in spatial analyses.
• The data can be used in economic, demographic and environmental reconstructions, e.g. to estimate past anthropopressure related to settlement, agriculture and forestry. It can also be helpful in assessing human interference with river ecosystems.
• The point locations of mills can be helpful in identifying areas where the local population used traditional knowledge of the natural environment, and thus in the contemporary assessment of the natural environment for renewable energy sources.
• The data on the remains of former mills can be suitable for the protection of cultural heritage sites as technical monuments related to traditional food processing and industry and for the protection of places of natural value.

## 1 Data Description

Mills with various types of propulsion, especially natural ones, have played an important role in the cultural landscape of many regions of the world for many centuries [1], [2], [3], [4], [5]. Their presence is associated not only with the processing of agricultural produce, wood, fabrics, and paper, but also affects various natural and social processes, such as water retention and changes in the water network (e.g. millraces) [6], changes in the relief (e.g. dikes, ditches) [7], creating and maintaining important habitats for aquatic organisms [8], and activating local communities [9]. For the northern part of Poland, a cultural landscape typology was prepared based on mills [10], and the presence of mills could be an important manifestation of socio-economic development [11]. Old mills still attract attention due to the high potential of landscape ecosystem services [12], [13], [14], and due to the need to preserve their remains as cultural heritage or natural valuable areas. The data on the mills is usually reconstructed using old maps, but also other historical sources of information such as sketches, drawings, inventories, sometimes also thanks to field research [10,15].

We used two sets of historical maps to identify the locations of the old mills. The first was 53 sheets of the 1880 administrative map, 1:115,200 scale, and the second one was 109 sheets of the military topographic map, 1:100,000 scale, from the 1920s–1930s.

Our data contains two point layers and six grid layers (10 km side squares). All data is available in an open shapefile, GML, GeoJSON, KML/KMZ formats, commonly used in Geographic Information Systems [16].

**Files: mills_1880_GAL; mills_1920_1930_GAL**

Point layers contain the following attributes for each of the mills: auto-numbered numeric identifier (FID), geometry type (Shape), mill type (Type), map sheet date (Map_year), longitude (Long), and latitude (Lat).

The “Map_year” attribute is the same for the entire 1880 set and is 1880.

**Fig. 1:**
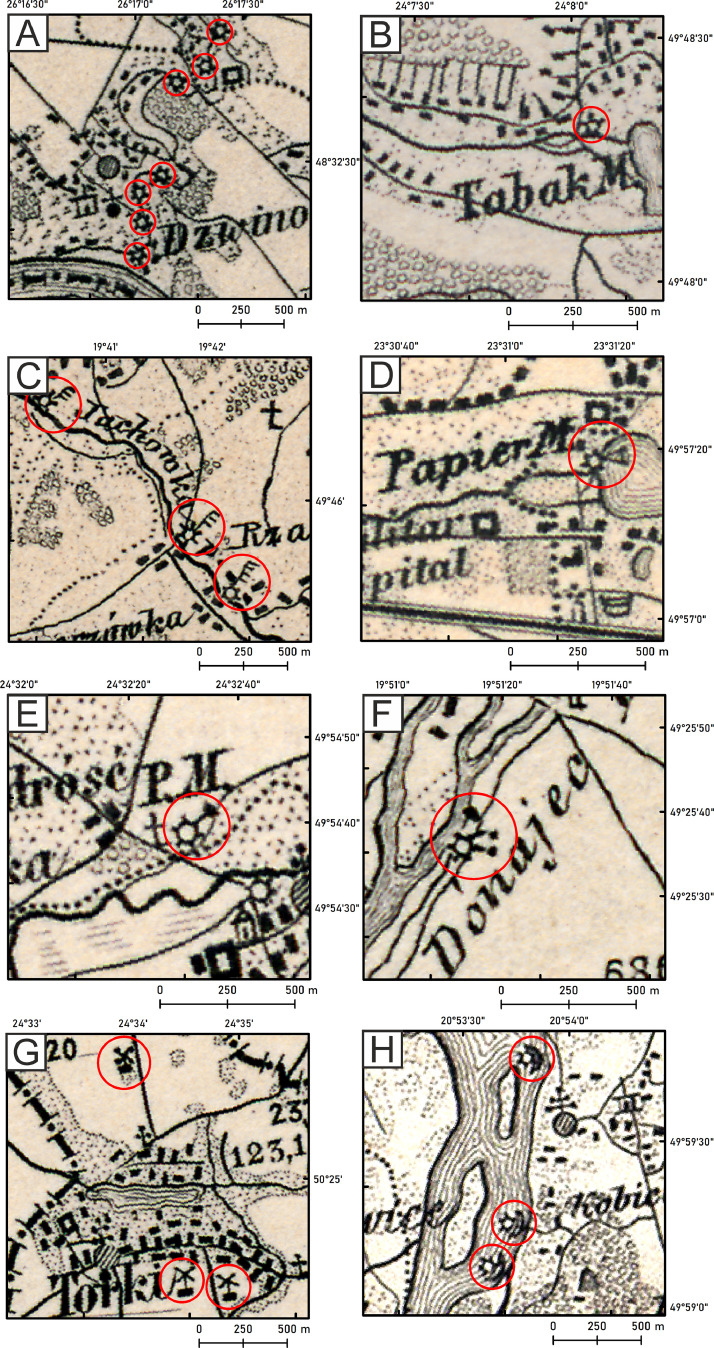
Types of mills for 1880. A – gristmills, B – gristmill (snuff mill, *ger*. Tabak M. inscription), C – sawmills, D – paper mill (*ger*. Papier M. inscription), E – powder mill, F – fulling mill, G – windmills, H – ship mills.

According to the legend of these maps and explanations, the following types of mills can be distinguished for 1880 (Fig. 1):

1. Gristmill (*ger*. Fruchtmühle),
2. Sawmill (*ger*. Sägemühle),
3. Paper mill (*ger*. Papiermühle),
4. Powder mill (*ger*. Pulvermühle),
5. Fulling mill (*ger*. Walkmühle),
6. Windmill (*ger*. Windmühle),
7. Ship mill, (*ger*. Schiffmühle).

**Fig. 2:**
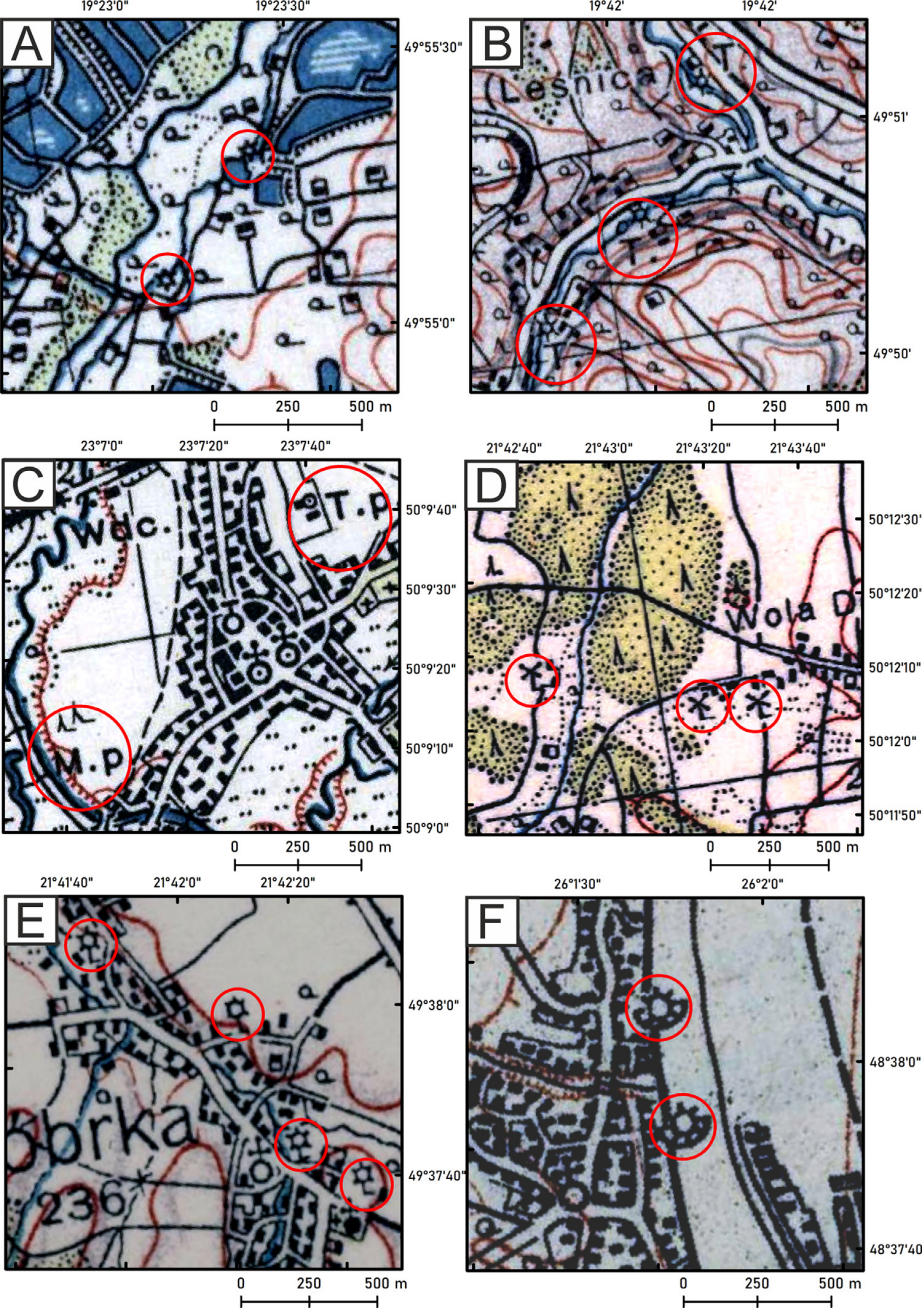
Selected types of mills for 1920s–1930s. A – watermills, B – sawmills with water wheel, C – steam sawmill with T. p inscription and steam mill with M. p inscription, D – windmills, E – wind turbines, F – ship mills.

For the 1920s–1930s, the following types of mills were distinguished according to the legend of these maps and explanations (Fig. 2):

1. Watermill,
2. Steam mill,
3. Sawmill,
4. Sawmill with water wheel,
5. Motor sawmill,
6. Steam sawmill,
7. Steam mill,
8. Windmill,
9. Wind turbine,
10. Ship mill.

The “Map_year” attribute for mills discovered on the interwar maps ranges across the years from 1922 to 1939. We took into account the years of checking the map content in the field, and if they were not given, we indicated the years of the cartographic document. For most map sheets, they are at least one year earlier than their release dates.

**Files: gristmills_1880_GAL_GRID; gristmills_1920_1930_GAL_GRID; sawmills_1880_GAL_GRID; sawmills_1920_1930_GAL_GRID; windmills_1880_GAL_GRID; windmills_1920_1930_GAL_GRID**

**Fig. 3:**
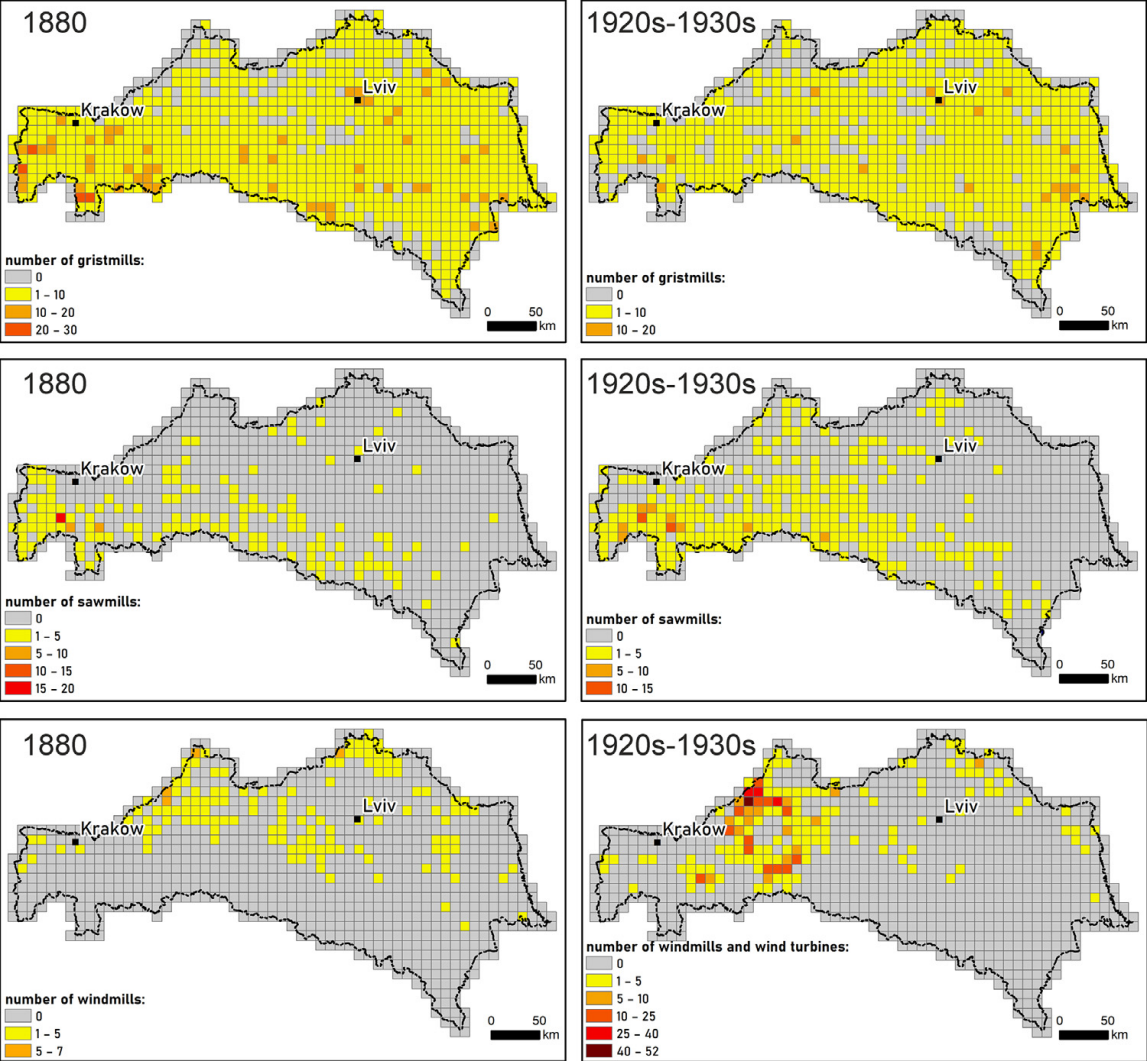
Aggregated mill types counted in a 10 km reference grid for 1880 and 1920s–1930s.

A reference grid designed by the European Environment Agency (EEA) was used to create the grid layers, consisting of cells with sides of 10 km. In the set we provide, it contains the following attributes: auto-numbered numeric identifier of the cell (FID), geometry type (Shape), cell code (CellCode), east (EofOrigin) and north (NofOrigin) cell start coordinates, and an attribute (Count) in which aggregated mill types are counted for each cell: gristmills, sawmills, windmills (Fig. 3).

## 2 Experimental Design, Materials and Methods

**Fig. 4:**
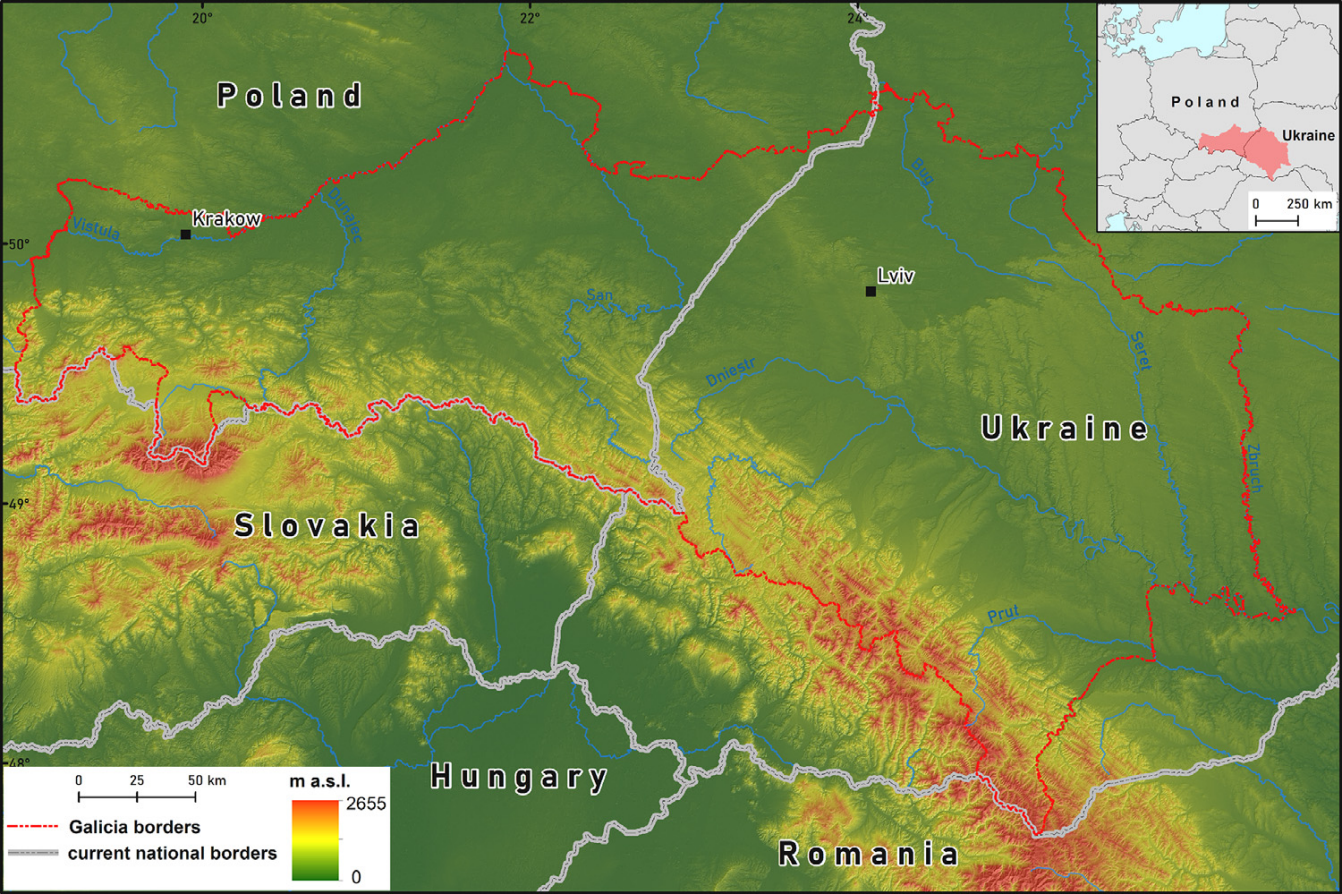
Map of the study area.

The data collection area is Galicia, 78,500 km^2^, currently in Ukraine and Poland. The Polish-Lithuanian Commonwealth (the Crown of the Kingdom of Poland and the Grand Duchy of Lithuania) at the end of the 18th century was divided by the Russian Empire, the Kingdom of Prussia and the Habsburg Monarchy, from 1867 the Austro-Hungarian Empire. Galicia covers the territories occupied by Austria, and its borders were shaped after the Congress of Vienna in 1815, the inclusion of the Republic of Kraków in 1846 and the exclusion of Bukovina in 1849. Within such borders (Fig. 4), Galicia, as a crown land, existed until World War I, and after Poland regained independence in 1918, Galicia became a part of it until the World War II. After the World War II, most of its territory remained within the borders of the Soviet Union (Ukrainian Soviet Socialist Republic), and since 1991, independent Ukraine.

We used two sets of historical maps.

**Map 1.** The first is an administrative map covering the Kingdom of Galicia and Lodomeria together with the Grand Duchy of Kraków and the Duchy of Oświęcim, Zator and Bukovina (*ger*. Administrativ-Karte von den Königreichen Galizien und Lodomerien mit dem Grossherzogthume Krakau und den Herzogthümern Auschwitz, Zator und Bukowina in 60 Blättern), 1:115,200 scale from 1880. This map is known informally as the Kummerer Ritter von Kummersberg map, after the Austrian military man who developed the map. It is a version of the map from 1855 updated on the basis of the maps of the Military Geographical Institute in Vienna (*ger*. k.u.k. Militärgeographisches Institut, MGI). Scans with a resolution of 600 dpi were obtained from the University of Vienna in TIFF format, without georeferencing. The original map projection is unknown but probably is based on cadastral maps in the scale 1:2880 in the Cassini-Soldner projection. The maps were georeferenced by us to the LAEA projection (EPSG 9820) in ArcMap 10.8 (“Georeferenced” tools). Geometric correction and georeferencing was obtained using 2nd-order polynomial transformation. For map sheets with small amounts of coverage along the Galicia borderland, a 1st-order polynomial transformation was applied. Maps of the second military survey in the scale 1:28,800 served as the basis for the georeferencing [17]. For 53 map sheets covering Galicia, 2498 control points were used and the root mean square error was 87.01 m. During vectorisation, we found another type of mill, not explained in the legend. For the geographic coordinates of these mills, the second military survey maps shows the signatures of the mills described as ship mills [18].

In the map from 1880, in the case of five types, the mills were classified according to their purpose, raw material or product. Water mills are divided by construction method, and windmills by the type of propulsion. The Gristmill type is defined more broadly than processing of cereals and refers to agricultural produce in general, e.g. processing of tobacco (snuff mills) or hops (breweries).

**Map 2.** The second source for the reconstruction of how mills were distributed is the topographic map of the Military Geographical Institute (Tactical Map of Poland) in a scale of 1:100,000. The area of Galicia is covered by 109 sheets. It was made in independent Poland, mainly in the 1930s, but sheets from the 1920s are also available for some areas. These maps were assessed as a reliable source of information in geographic and historical research of the period before the World War II [19]. The map sheets were obtained from the website mapywig.org, which identifies the following sources of scans: the Jagiellonian Digital Library (for 103 sheets), Library of Congress (for five sheets), and the Map Storehouse of the Earth Sciences Library of the University of Silesia (one sheet). The scans of the maps had a resolution of 600 dpi and were in .jpeg format. The original map projection was based on unified quasi-stereographic projection with a central point in Borowa Góra observatory. We have georeferenced all the sheets based on Polish topographic maps at a scale of 1:25,000 from the 1970s and 1980s, and modern World Imagery high-resolution satellite images, especially helpful for the area of Ukraine. Georeferencing was obtained using 2nd-order polynomial transformation.The LAEA (EPSG 9820) was adopted as the reference system. 5167 checkpoints were used, and the RMS error was 25.24 m.

According to the legend and technical instructions of this map, ten types of mills can be distinguished. Technical progress and electrification led to the emergence of new types of mills compared to 1880.

**Data acquisition.** Manual vectorisation of the mills on the computer screen was carried out using the ArcMap 10.8 software (“Edit” tool). At the points of the main cartographic symbols, a dot was placed indicating the mill location. In cases where there was only an inscription denoting the mill, a dot was placed on the inscription, or, if the inscription clearly referred to the building, the dot was placed on the building. A screen zoom of 1:4000–1:10,000 was used. The mill type attribute was assigned for each point, from a previously prepared database domain, in accordance with the legend and map instructions.

The grid layers were developed by us to allow comparing the mills’ locations for both periods due to different RMS errors. The grid layer, designed by the European Environment Agency (EEA) in the LAEA projection (EPSG 9820), was trimmed to the administrative boundaries of Galicia and joined spatially (Join Data based on spatial location) with the point layers of the mill locations. The Count option was used, counting points within each cell (a square with sides of 10 km)

**Validation.** Point data was verified in ArcMap topology (“Data Reviewer” tools) by Duplicates rule.

The number of mills acquired by us can be compared with the studies of historians and statisticians, although data for the same period is not always available or the period is generalised. For example, for 1886, 3,439 mills for districts in Galicia [20] (Table 1), compared to 3797 mills vectorised by us for 1880. At the end of the 19th century, windmills accounted for 3% of all mills [21], compared to 6% according to our data. For the interwar period [21], the percentage of windmills versus mills was at 4.6%, compared to 20.9% vectorised by us and respectively: water mills – 75% compared to 71% according to our data, motor mills – 15.3% compared to 3% vectorised using maps.

**Table 1:** Comparison of the number of mills for selected districts in the western and eastern part of Galicia.

		Number of mills	
District name	Part of Galicia	for 1880 from maps	for 1886 from H. Kramarz
Biała	western	96	97
Borszczów	eastern	66	8
Dolina	eastern	92	91
Drohobycz	eastern	63	64
Myślenice	western	65	59
Nowy Targ	western	157	204
Stryj	eastern	131	135
Wadowice	western	80	77
Zaleszczyki	eastern	61	72
Żywiec	western	100	36

## Ethics Statement

The authors declare that the manuscript meets all the rules and conditions described in the ``Ethics in publishing'' section (https://www.elsevier.com/journals/data-in-brief/2352-3409/guide-for-authors). During the dataset gathering, no experiments on humans nor animals were involved.

## CRediT authorship contribution statement

**Krzysztof Ostafin:** Conceptualization, Writing – original draft. **Magdalena Jasionek:** Writing – review & editing. **Dominik Kaim:** Writing – review & editing. **Anna Miklar:** Writing – review & editing.

## Declaration of Competing Interest

The authors declare that they have no known competing financial interests or personal relationships that could have appeared to influence the work reported in this paper.

## References

[1] Grano M.C., Del Monte M., Lazzari M., Bishop P. (2016). Fluvial dynamics and watermills location in basilicata (southern Italy). Geogr. Fis. Dinam. Quat..

[2] Limmer A., Zumbrägel C. (2020). Waterpower romance: the cultural myth of dying watermills in German hydro-narratives around 1900. Water Hist..

[3] Cherry J., Rothenberg M. (2021). Costly signaling and windmill-building: inter-island technological variability on eighteenth-century sugar estates in the lesser antilles. Int. J. Hist. Archaeol..

[4] Mishmastnehi M., Milke R., Bernbeck R. (2021). A forgotten technology: the production of artificial millstones for windmills in Sistan, southeastern Iran. J. Archaeol. Sci..

[5] Jagxhiu B., Çadraku H. (2021). Identification and restoration of the traditional watermills in Lipjan. Pollack Period..

[6] Witkowski K., Witkowski M. (2018). the Impact of Watermills on Changes in the Hydrographic Network in the Carpathian Foothills in Poland. Carpathian J. Earth Environ. Sci..

[7] Podgórski Z. (2009). Młyny wodne w krajobrazie Pojezierza Chełmińskiego. Pr. i Stud. Geogr..

[8] Wood P.J., Barker S. (2000). Old industrial mill ponds: a neglected ecological resource. Appl. Geogr..

[9] Hognogi G.G., Marian-Potra A.C., Pop A.M., Mălăescu S. (2021). Importance of watermills for the Romanian local community. J. Rural Stud..

[10] Brykała D., Podgórski Z. (2020). Evolution of landscapes influenced by watermills, based on examples from Northern Poland. Landsc. Urban Plan..

[11] Kargol T. (2010). Ziemiaństwo a uprzemysłowienie Galicji na przełomie XIX i XX w. (do 1918 r.). Stud. z Hist. Społeczno-Gospodarczej.

[12] Muller G., Kauppert K. (2002). Old watermills—Britain's new source of energy?. Proc. Inst. Civ. Eng. Civ. Eng..

[13] O. Paish, R. Armstrong-Evans, R. Saini, D. Singh, D. Kedia, The development of traditional Himalayan watermills for sustainable village-scale micro-hydropower, in: 2020: pp. 337–349, doi:10.1201/9781003078029-34.

[14] Brykała D., Podgórski Z., Sarnowski Ł., Lamparski P., Kordowski J. (2015). Wykorzystanie energii wiatru i wody w okresie ostatnich 200 lat na obszarze województwa kujawsko-pomorskiego. Pr. Kom. Kraj. Kult..

[15] Franczak P., Listwan-Franczak K. (2019). Ślady dawnych siłowni wodnych zapisane w rzeźbie terenu i krajobrazie doliny Skawicy. Aura.

[16] Ostafin K., Jasionek M., Kaim D., Miklar A. (2021). Historical dataset of mills for Galicia in the Austro-Hungarian Empire/southern Poland from 1880 to the 1930s. Mendeley Data.

[17] Ostafin K., Kaim D., Troll M., Maciejowski W. (2020). The authorship of the Second Military Survey of Galicia and Austrian Silesia at the scale 1:28,800 and the consistency of sheet content based on selected examples. Polish Cartogr. Rev..

[18] J. Zaffauk, Signaturen in-und ausländischer Plan-und Kartenwerke: nebst Angabe der in Karten und Plänen am häufigsten vorkommenden Worte und Wort-Abkürzungen in 12 Sprachen; zum Plan-und Kartenlesen; prämiirt vom internationalen geographischen Congress in Venedig; mi, Hölzel, Wien, 1889.

[19] Kuna J. (2018). Partially compiled’ maps 1:25,000 by Polish Military Geographical Institute (1919–1939). Polish Cartogr. Rev..

[20] Kramarz H. (2008). Galicyjski Związek Młynów we Lwowie jako stowarzyszenie przedsiębiorców. Res Gestae. Czasopismo Historyczne.

[21] B. Baranowski, Polskie młynarstwo (Polish milling), Zakład Narodowy im. Ossolińskich, Wrocław, Warszawa, Kraków, Gdańsk, 1977.

